# Children’s Effortful Control Skills, but Not Their Prosocial Skills, Relate to Their Reactions to Classroom Noise

**DOI:** 10.3390/ijerph19148815

**Published:** 2022-07-20

**Authors:** Jessica Massonnié, Philippe Frasseto, Terry Ng-Knight, Katie Gilligan-Lee, Natasha Kirkham, Denis Mareschal

**Affiliations:** 1 Centre for Brain and Cognitive Development, Department of Psychological Sciences, Birkbeck, University of London, Malet Street, London WC1E 7HX, UK; n.kirkham@bbk.ac.uk (N.K.); d.mareschal@bbk.ac.uk (D.M.); 2 School of Education and Sociology, Faculty of Humanities and Social Sciences, University of Portsmouth, St. George’s Building, 141 High Street, Portsmouth PO1 2HY, UK; 3 Académie de Corse, Corsica, 20200 Bastia, France; philippe.frasseto@gmail.com; 4 School of Psychology, Faculty of Health and Medical Sciences, University of Surrey, Guildford GU2 7XH, UK; terryknight1983@hotmail.com (T.N.-K.); k.gilligan@surrey.ac.uk (K.G.-L.)

**Keywords:** noise, noise sensitivity, health, children, classroom, attention, distraction, annoyance, temperament, prosocial skills

## Abstract

Environmental noise is one of the main sources of pollution in today’s modern world. Health effects associated with noise depend on both environmental exposure and individuals’ noise sensitivity. However, still little is known as to why some children are more noise sensitive than others. Studies to date have focused on adult populations and have not considered both cognitive and personality factors when explaining noise sensitivity. The current research investigates individual differences in noise sensitivity among elementary school children, with the aim of shedding light on its underlying mechanisms. Study 1 (*n* = 112) validated a novel questionnaire assessing children’s reactions to classroom noise against two measures of noise sensitivity that are commonly used in adult populations. Study 2 (*n* = 237) investigated how children’s reactions to classroom noise covaried with their effortful control and prosocial skills, both measured through a teacher report. Prosocial skills were not related to children’s reactions to noise. However, children with lower effortful control skills reported more negative reactions to classroom noise. Given the importance of effortful control skills to succeed at school, children at risk of school difficulty might also be the ones who are particularly vulnerable to noise.

## 1. Introduction

More than 100 million people in Europe are exposed to harmful levels of environmental noise, or in other words, to unwanted sounds exceeding the thresholds recommended by the World Health Organisation [1]. A total of 22 million people experience high annoyance towards noise, and 6.5 million suffer from noise-related sleep disturbances [1]. Noise annoyance and sleep disturbances have been proposed as the two main causes for the negative long-term health effects of noise in adults [1]. Each year, noise is estimated to cause 12,000 premature deaths and to induce learning impairment in 12,500 school children [1]. However, not everyone suffers from noise to the same extent. Adults who are more sensitive to noise are more likely to be annoyed by noise and to suffer from it. Overall, they are more likely to suffer from insomnia, stress, anxiety and depression [2,3,4,5] and to have a lower quality of life [6]. The psychological mechanisms underlying noise sensitivity and potentially its association with mental health difficulties are still unclear. This is particularly true when working with children. To understand the impact of noise on children’s health more fully, we need (1) a clear definition of noise sensitivity, (2) putative mechanisms for noise sensitivity, (3) an investigation of individual differences which link to such mechanisms.

### 1.1. Defining and Measuring Noise Sensitivity

Noise sensitivity describes the internal states of an individual (be they physiological, psychological, related to the individual’s life style or to the activities they are engaged in) that increase their degree of reactivity to noise in general [7]. Noise sensitivity reflects general reactions to noise. It contrasts with noise annoyance, which reflects a reaction to a specific source of noise (e.g., aeroplanes, traffic). ‘Reactivity’ refers to negative, emotional reactions such as annoyance, anger, depression or frustration [7].

Noise sensitivity can be measured by four main types of questionnaires: (1) single-item scales assessing to what extent respondents are sensitive to noise [3] or disturbed by noise in general [8]; (2) multiple-item questionnaires assessing subjective reactions to noise in a variety of settings (i.e., the home, office, and/or study environment [9,10,11]); (3) multiple-item questionnaires assessing subjective reactions to noise in one specific setting (i.e., the office [12,13], the school environment [14,15]); (4) single-item questionnaires assessing subjective reactions towards a specific source of noise (i.e., aircraft noise, traffic noise [16,17]).

Individuals’ scores on these different noise sensitivity measures are typically correlated [5,13], but each have specific advantages and disadvantages. Single-item measures are quick to administer and can be used in large scale surveys. However, they are very general and can be interpreted in different ways by respondents. Multiple-item measures, such as the one used by Weinstein (1978) [11], are more naturalistic and refer to real-life situations. They include various reactions to noise such as annoyance (e.g., ‘I get annoyed when my neighbours are noisy’), disturbance (e.g., ‘When I want to be alone, it disturbs me to hear outside noises’), and loss of focus (e.g., ‘Even music I normally like will bother me if I am trying to concentrate’) across different contexts. The issue with questionnaires that refer to various reactions to noise across different contexts, as in the previous examples, is that it is difficult to understand if the same reactions hold across situations: Is the person equally annoyed in the presence of noise when watching a movie and reading? Such questionnaires also raise the question of how comparable different reactions are in the same context: Are feeling annoyed and lacking focus when reading the same thing?

Questionnaires that focus on a specific environment, such as the office, as studied by Kjellberg et al. (1996) [12], or the school, as studied by Massonnié et al., 2020 [15], reveal the multi-faceted nature of people’s reactions to noise. They show that distraction and annoyance are two distinct yet correlated reactions to noise. Distraction relates to a process of interference, or difficulty achieving goals in the presence of noise. It does not relate to an emotional reaction in and of itself. Noise annoyance, however, involves a negative emotional reaction that might or might not arise following distraction from noise, depending on participants’ judgements and attitudes [18,19].

### 1.2. Candidate Mechanisms Underlying Noise Sensitivity 

Research on the mechanisms underlying noise sensitivity in adults have typically used different measures of noise sensitivity without systematically dissociating different reactions to noise.

Early studies from the 1980s onwards have investigated basic physiological and sensory processes. Results that relate noise sensitivity with participants’ heart rate, blood pressure, skin conductance and the level at which they start to find noise uncomfortable, are inconsistent [20,21,22,23]. Furthermore, adults with high and low sensitivity to noise do not differ in terms of absolute auditory thresholds or capacity to discriminate between tones of different intensities [22,24,25]. Overall, psychoacoustical factors account for around 15% of the inter-individual variance in noise sensitivity [25]. This leaves room for other explanatory factors. 

More recent studies have shifted the focus of investigation from the peripheral auditory system to central processes [26,27]. Noise-sensitive individuals seem to have difficulties encoding auditory information and discriminating changes in sound noisiness (i.e., changes in the sound’s waveform [28]). They may have reduced sensory gating; that is to say, difficulties inhibiting or filtering unnecessary sensory inputs [29]. Dealing with the negative emotions associated with noise might also tax attentional resources [30]. These results hint at the role of attentional factors underlying noise sensitivity. 

Finally, personality traits play a role in how people react to and interpret noise. Neuroticism (the tendency to be emotionally unstable, to feel tense, nervous, worried, or anxious) and introversion have been associated with higher noise sensitivity [4,31,32]. Neurotics and introverts may have lower arousal thresholds and, as such, would be more easily stressed and overwhelmed by noise, with anxiety or worries making it difficult to perceive the noise as positive [33]. Extraverts, on the contrary, seek more external stimulation and arousal. They may perceive noise as a social signal, and given their lower need for privacy, may have more positive and less stressful attitudes toward noise [11,32]. More specifically, Weinstein (1978) noted that participants who reported lower sociability, tolerance and social presence were more sensitive to noise. However, in one group of participants, socialisation tendencies were positively associated with noise sensitivity. This raises the question as to whether prosocial behaviour, in general, is related to noise sensitivity. 

To date, no research has considered personality and cognitive factors in combination in explaining noise sensitivity. Furthermore, research investigating the mechanisms underlying noise sensitivity has failed to connect with research highlighting different types of reactions to noise. Little is known about the mechanisms underlying different reactions to noise, such as distraction and annoyance, especially among children.

### 1.3. Research on Children

Key studies investigating the impact of noise on children have compared children from comparatively quieter and noisier neighbourhoods, establishing a connection between children’s exposure to environmental transportation noise and stress, annoyance, lower psychological quality of life, reading difficulties and attentional difficulties [16,17,34,35,36]. These studies have raised awareness on the issue of noise for communities, but do not contribute to understanding underlying mechanisms because the children being compared with each other might differ both in terms of their noise environment and individual characteristics. We therefore need studies investigating interindividual differences in noise sensitivity among children from the same environment. In experimental conditions, extravert children tolerate higher levels of experimentally induced white noise than introverts [37], but it is unclear how these results would generalise to naturalistic classroom environments.

The classroom offers one example of a common environment in which children with diverse sensitivities are exposed to similar sources of noise. Researchers have investigated children’s perceptions of sources of noise in the classroom [38], factors influencing children’s reactions to noise in the classroom [14], children’s perceived effort, tiredness, disturbance and irritation when performing specific tasks in the presence of noise [39,40]. Regarding individual differences, no differences in prosocial behaviour, conduct problems, hyperactivity, emotional symptoms and peer problems were found between schoolchildren reporting high and low annoyance towards airport noise [41]. However, the comparison was dichotomised with only twenty-two pupils in the ‘high annoyance’ group, which reduced power. Parental reports revealed that elementary and middle school pupils who are more noise sensitive also have more behavioural problems [42]. However, noise sensitivity measures were not completed by the children themselves and therefore might not accurately reflect their perspectives. 

With regard to cognitive differences, in a recent study on elementary school pupils by Massonnié et al., 2020, children who reported more interference and annoyance from noise also reported greater difficulties in switching from one task to another, and a greater propensity for mind-wandering [15]. These results support the idea that attentional control underlies reactions to noise, because both mind-wandering and switching skills rely on complex attentional processes, allowing individuals to purposefully stay on task [43,44,45]. However, to our knowledge, no study has explicitly investigated the associations between children’s ability to regulate their attention and their subjective reactions to noise. It is fundamental to bridge this gap in order to understand which children might suffer more from noise than others, and to offer protective solutions. The current research investigates individual differences in noise sensitivity among elementary school pupils, with a focus on prosocial behaviour and attentional skills.

### 1.4. Aims of the Research 

Study 1 sets up the methodological background necessary for measuring noise sensitivity in children. Based on a sample of 112 French elementary school pupils, it further validated the questionnaire used by Massonnié et al., 2020. This questionnaire distinguished hearing difficulties, annoyance, attention capture and interference from classroom noise [15]. In Study 1, it was validated against (1) a single-item measure of noise sensitivity, and (2) Weinstein (1978)’s multiple-item questionnaire of noise sensitivity, assessing reactions to noise in a variety of everyday life situations [11]. All the measures were expected to be moderately correlated with one another, as found in adult populations. Annoyance reactions were expected to be the most strongly correlated with the single-item measure of noise sensitivity because noise sensitivity is conceptualised as triggering negative emotional reactions in the presence of noise [7].

Using the Massonnié et al. (2020) questionnaire, which was newly validated in Study 1, Study 2 tested whether elementary school pupils’ reactions to classroom noise were associated with their attentional skills and personality, with a focus on effortful control and prosocial behaviour. Effortful control refers to temperament-based differences in reactivity and self-regulation [46]. It includes children’s capacity to (1) focus their attention (Attentional Focus), (2) manage their impulsivity, (3) suppress inappropriate actions or responses (Inhibitory Control), and (4) perform an action when they have a strong tendency to avoid it (Activation Control) [47]. We predicted that prosocial behaviour, and better self-regulation, would be associated with less negative reactions to classroom noise [11,15,32].

## 2. Study 1

### 2.1. Participants, Measures and Procedure

#### 2.1.1. Participants

Neurotypical children in their last two years of elementary school (equivalent of Year 5 and Year 6 in the United Kingdom, or 4th and 5th Grade in the United States) were recruited from six French classrooms in Corsica. The classrooms were situated across both urban and suburban areas. The classrooms were only moderately exposed to external environmental noise, and the noise levels generated inside the classroom typically covered the sound that could be heard from the outside (more information on noise measurements can be found in [48]). Parental consent was obtained for 113 pupils (52 Year 5s, and 61 Year 6s). One classroom additionally contained eight children in Year 4 who participated in the research project, but their data were excluded from the current analyses for the purpose of homogeneity. Data from another child, for whom a hearing disorder was reported by the parents, were also excluded from the analyses. The final sample included 112 pupils, from 8.70 to 11.38 years of age (*M* = 10.03; *SD* = 0.60; 48.2% female). 

#### 2.1.2. Institutional Review Board Statement

All the participants gave verbal consent to participate, and written informed consent was obtained from their guardian. Both the parents and children were provided with an information sheet explaining the purpose of the study and explaining the principles behind data anonymity. Participants were informed of their right to withdraw from the study at any time without any reprisal. The study was conducted in accordance with the Declaration of Helsinki and received ethical approval from the Ethics Committee of the Department of Psychological Sciences of Birkbeck, University of London (Reference Number: 161773; Date of Approval: 21 September 2017). The six participating classrooms were under the jurisdiction of a French educational inspector who approved the ethical guidelines of the study. 

#### 2.1.3. Measures

All measures were part of a larger study investigating the impact of sound awareness and mindfulness interventions on classroom noise levels, children’s reactions to noise, attentional skills, working memory and school performance [48]. All the measures reported here were carried out at baseline, i.e., before the interventions. All the questionnaires were completed in French but have been translated into English for the purpose of publication (see Appendix A).

##### Noise Sensitivity (Single Item)

Pupils were asked how sensitive they were to noise, in general. They replied on a 4-point response scale: (1) Not sensitive at all, (2) A bit sensitive, (3) Rather sensitive, (4) Very sensitive.

##### Noise Sensitivity (Multiple Items)

Weinstein’s (1978) questionnaire on noise sensitivity [11], which has previously been validated with young adults, was adapted for use with elementary school children. The original measure included 21 items. Items that were inappropriate to use with children (e.g., ‘I would not mind living on a noisy street if the apartment I had was nice’) were excluded. The item, ‘I am sensitive to noise’, was also excluded because it corresponded to the single-item measure of noise sensitivity. The final adaptation resulted in the selection of seven items (corresponding to items 4, 7, 10, 11, 16, 18 and 19 from Weinstein’s (1978) questionnaire [11]). These seven items were piloted on 15 children in Year 5 before being used in the current study. Children indicated to what extent each of the statements was true for them, by responding using a 4-point response scale: (1) Not at all true, (2) A bit true, (3) Rather true, (4) Definitely true. An exploratory factor analysis was used to analyse the factorial structure of the questionnaire. The Keyser–Meyer–Olkin measure of sampling adequacy was good (.73). Bartlett’s test for sphericity was significant (*X*^2^(21) = 79.37, *p* < .001), indicating that inter-item correlations were sufficient. These inter-item correlations ranged from .10 to .40 with an average of .22. One factor had an Eigenvalue greater than 1 and accounted for 33.77% of the overall variance. All the items had a factor loading above .45. The reliability of this scale was fair (α = .67) [49]. A single score was therefore extracted from this questionnaire.

##### Children’s Reactions to Classroom Noise 

This study aimed to further validate the Massonnié et al. (2020) questionnaire assessing children’s reactions to classroom noise [15]. The questionnaire included seventeen items across five dimensions (factorial analyses are published in [15]): (1) Children’s perception of noise levels in the classroom (e.g., ‘Do you think your classroom is noisy?’, three items, α = .80); (2) Hearing difficulties (e.g., ‘It’s hard to hear what the person says’, two items, α = .73); (3) Annoyance from noise (e.g., ‘You are annoyed by noise in the classroom’, five items, α = .86); (4) Attention capture (the fact that children notice noise, e.g., ‘Classroom noise attracts your attention’, three items, α = .90) and (5) Interference (the fact that noise catches children’s attention and interferes with their ongoing task, e.g., ‘If noise catches your attention you lose track of the discussion’, four items, α = .81). Questions about annoyance, attention capture and interference were asked about four different classroom situations: (1) when the teacher, or a classmate, talked to the entire classroom; (2) when the teacher, or a classmate came closer to talk to the child; (3) individual work; (4) group work (the question about attention capture from noise during group work was not used in the analyses given that factorial analyses revealed unsatisfactory factor loadings [15]). Questions about hearing difficulties were only asked for the first two situations. For all questions, children answered on a 4-point response scale: (1) Almost never, (2) Rarely, (3) Quite often, (4) Very often. Five sub-scores were extracted from the questionnaire, corresponding to each of the five dimensions.

##### Mood

Children were asked three questions about how (1) Calm, (2) Relaxed and (3) Irritated they were in the present moment. They answered on a 4-point response scale with: (1) Not at all, (2) A bit calm, (3) Rather, (4) Very (calm/relaxed/irritated). The reliability obtained when grouping the items in the same scale was only moderate (α = .60) and the Keyser–Meyer–Olkin measure of sampling adequacy was bad (.56), so the three items were kept separate.

#### 2.1.4. Procedure

Children filled in the paper-based questionnaires in their usual classroom, in a group session and under the supervision of their teacher. Two versions of the questionnaire were created. Version A presented the questions in the following order: (1) children’s perception of noise levels in the classroom; (2) noise sensitivity (single item); (3) noise sensitivity (multiple items); (4) mood and finally (5) hearing difficulty, attention capture, interference and annoyance from noise. The order was reversed in Version B. Half of the children were given Version A and half of the children were given Version B to ensure that there were no order effects.

### 2.2. Results

#### 2.2.1. Descriptive Statistics

In total, 8.41% of data points were missing. This was due to children being absent at the testing session, or not replying to a given item. Little’s MCAR test was non-significant (*χ*^2^(501) = 521.99, *p* = .250) [50], indicating that the data were missing completely at random. The average of the items constituting a given subscale was calculated for every child who had no more than one missing item at this given scale. Descriptive statistics are reported in Table 1. 

A visual analysis of the distributions showed that several variables deviated from a normal distribution. The distribution for the single-item measure of noise sensitivity was positively skewed, with 81.6% of the children saying they were not at all or only a bit noise sensitive; and so was the case for the item assessing hearing difficulties (48.1% of the children almost never experienced hearing difficulties). In addition, 82.4% of the children said that they did not feel irritated at all. Eighty percent of the children said that they were rather calm or very calm, the distribution for this variable being negatively skewed. A few participants deviated from these general patterns. In particular, three children reported that they very often experienced hearing difficulties, and one child reported being very irritated. 

#### 2.2.2. Correlations

Before running inferential statistics, each child’s score was centred on the classroom’s mean. This procedure allows us to obtain unbiased estimates at the individual level [51,52] when observations are not independent (children being nested within classrooms). This procedure also resulted in variables following a normal distribution, and thus satisfied the assumptions of Pearson’s correlations. These are reported in Table 2.

The single-item and multiple-items measures of noise sensitivity were moderately and positively correlated with each other. The four reactions to noise (hearing difficulties, attention capture, interference and annoyance from classroom noise) were all moderately too strongly positively correlated with each other, and with an overall feeling of irritation. Both measures of noise sensitivity were associated with greater hearing difficulties, distractibility and annoyance from noise in the classroom. The strongest association between children’s noise sensitivity and their reactions to noise was found for the subscale of annoyance. In addition, the multiple items measure of noise sensitivity was positively associated with the perception of noise in the classroom and with attention capture from noise. This was not the case for the single-item measure of noise sensitivity. No measures of noise sensitivity were significantly correlated with mood, with the exception of the multiple items measure being significantly and positively correlated with children feeling irritated when they filled the questionnaire. Controlling for age did not change the overall pattern of associations.

### 2.3. Discussion

Study 1 demonstrated how a novel questionnaire assessing children’s reactions to classroom noise associates with questionnaires traditionally used with adult populations. The single-item and multiple-item measures of noise sensitivity were moderately correlated with each other. The multiple items measure of noise sensitivity, which represented concrete everyday life situations was consistently associated with the four reactions to classroom noise. The single-item measure of noise sensitivity, which was more general and abstract, was less consistently associated with children’s reactions to classroom noise. 

Each of the two measures of noise sensitivity was more strongly correlated with reactions of annoyance than with other types of reactions such as attention capture and interference from noise. This is in line with the conceptualisation of noise sensitivity as triggering negative emotions [7], and gives weight to the idea that annoyance and distraction (which indicates interference from noise) are related yet distinct constructs [12,15]. Based on these findings, general noise sensitivity measures might focus more on emotional reactions of annoyance than on cognitive processes of interference between noise and an ongoing task. 

One key limitation of Study 1 was the modest internal consistency of the multiple items measure of noise sensitivity [11]. This might have been due to the diversity of situations encompassed in the questionnaire, and to the fact that it had originally been designed for use with university students. Using the newly validated questionnaire from Massonnié et al. (2020), Study 2 investigated the associations between children’s effortful control, prosocial behaviour and their reactions to classroom noise.

## 3. Study 2

### 3.1. Participants, Measures and Procedure

#### 3.1.1. Participants

Participants were 240 children recruited from an elementary school in the South of England, in the United Kingdom. Children were attending classes between Years 3 and 6, equivalent to 2nd to 5th Grade in the United States. Data from three children were removed from the analyses: one child was partially deaf and the other two had been diagnosed with autism, a condition that can be associated with hyper- or hypo-reactivity to sensory input [53,54]. The final sample was composed of 237 children (47% female), from 7.38 to 11.27 years of age (*M* = 9.39; *SD* = 1.09). There were 52 Year 3s, 63 Year 4s, 62 Year 5s and 60 Year 6s. 

#### 3.1.2. Institutional Review Board Statement

The study was conducted in accordance with the Declaration of Helsinki and was approved by the University Ethics Committee of the University of Surrey (Reference Number: UEC/2018/110/FHMS; Date of Approval: 10 December 2018). Passive consent was obtained from parents who could opt out of their child participating in the study and data collection. This was because the study was integrated in the usual school curriculum. Children completed a consent form indicating that their participation was voluntary and that they could withdraw at any time. 

#### 3.1.3. Measures

All the measures were part of a larger study investigating the impact of Taekwondo on children’s self-control [55]. The measures used in Study 2 were collected after the intervention (post-test data) from children in both the control and intervention groups. The goal of Study 2 was to investigate how individual variability in effortful control skills and prosocial behaviour related to children’s reactions to noise, irrespective of the possible impact of the intervention. 

##### Reactions to Classroom Noise

Children’s reactions to classroom noise were assessed using the four subscales from the questionnaire used in Study 1, namely hearing difficulties (α = .56), annoyance (α = .82), attention capture (α = .81) and interference (α = .83) from classroom noise [15]. The questionnaire was translated from French to English by a native French speaker. It was then verified by an English native speaker, who adjusted the wording to make it more child friendly. The items used in Study 2 are reported in Appendix B.

##### Effortful Control

For each child, teachers completed 17 items adapted from the Temperament in Middle Childhood Questionnaire (TMCQ) [47] that assessed Attentional Focus (four items, e.g., ‘Pays attention’, ‘When working on an activity, has a hard time keeping their mind on it’; α = .93), Impulsivity (five items, e.g., ‘Usually rushes into an activity without thinking about it; α = .88), Inhibitory Control (five items, e.g., ‘Can stop him/herself from doing things too quickly’; α = .77) and Activation Control (three items, e.g., ‘Has a hard time working on an assignment they find boring’; α = .84). Answers were provided on a 5-point response scale from (1) Almost always untrue to (5) Almost always true. Six items out of the seventeen items measuring effortful control were reverse-scored as appropriate. 

##### Prosocial Behaviour

For each child, teachers completed the prosocial behaviour subscale from the Strength and Difficulties Questionnaire (SDQ) [56]. It contained five items assessing positive social skills (e.g., ‘Often volunteers to help others) to which teachers replied using a 3-point response scale: (1) Not true, (2) Somewhat true, (3) Certainly true (α = .87).

#### 3.1.4. Procedure

Children completed the reactions to classroom noise questionnaire in their usual classroom, in a group session, under the supervision of their teacher. The questionnaire was paper based. For the younger children, the questions were read aloud by the teacher. Teachers completed the effortful control and prosocial behaviour measures on a paper-based questionnaire, in their own time. 

### 3.2. Results

#### 3.2.1. Descriptive Statistics

In total, 4.27% data points were missing. This was due to children being absent at the testing session, or not replying to a given item. Little’s MCAR test was non-significant (*χ*^2^(698) = 694.90, *p* = .526), indicating that the data were missing completely at random [50]. The average of the items constituting a given subscale was calculated for every child who had no more than one missing item at this given scale. Descriptive statistics are reported in Table 3. 

A visual inspection of the data showed that teachers tended to report good effortful control skills and prosocial behaviour in children. The distributions for the attentional focus, inhibitory control, activation control and prosocial behaviour subscales were negatively skewed. In contrast, the distribution of scores for the impulsivity subscale was positively skewed. There was, however, variability in the answers, showing that teachers did not report good effortful control for all children. Children reported reactions to noise that varied across the range of possible scores. There was a bimodal distribution for the reaction of annoyance towards classroom noise, with a mode around two and another mode at four (which represented 13.5% of the answers). This means that more than one tenth of the pupils reported being very annoyed by classroom noise in a variety of classroom situations.

Before running inferential statistics, the average score for each variable was centred on the classroom’s mean to control for the fact that children were nested within classrooms. This procedure also resulted in variables following a normal distribution, and thus satisfied the assumptions of Pearson’s correlations. Correlations between all the variables used in Study 2 are reported in Table 4. 

#### 3.2.2. Correlations

Correlations between the scores on the four subscales measuring children’s reactions to noise were positive and ranged from moderate to strong. Scores on the four subscales measuring effortful control were also moderately to strongly correlated, in the expected direction: attentional focus, inhibitory control and activation control were positively correlated with each other, and negatively correlated with impulsivity. In addition, children rated as more prosocial were also rated as having better attentional focus, inhibitory control, activation control, and as being less impulsive. 

Children’s reactions to noise were, to a certain extent, associated with effortful control. Children reporting greater hearing difficulties, annoyance and interference from noise also had lower attentional focus, inhibitory control and activation control. Children reporting more annoyance and interference from noise were also more impulsive. Attention capture from noise was not related to any of the effortful control subscales. Finally, prosocial behaviour did not correlate with any reaction to noise.

#### 3.2.3. Multiple Regressions

To better understand which temperament variables were most strongly linked with children’s reactions to noise, three multiple regressions analyses were carried out with hearing difficulties, annoyance, and interference from noise entered successively as dependent variables (attention capture was not considered as a dependent variable because it did not correlate with any of the temperament variables). A hierarchical procedure was followed. Age was entered in the first step as a control variable. The four effortful control subscales were entered in the second step (prosocial behaviour was not entered as an independent variable because it did not correlate with any reaction to noise). In this second step, a stepwise procedure was used. This is because the four predictors were strongly correlated to each other, as shown in Table 1. This can induce issues of multicollinearity in regression models. Given that correlations between each effortful control subscale and each reaction to noise is reported in Table 1, the objective of the stepwise regression analyses was to select the predictor(s) which accounted for the most variance in the outcome variable. The analyses, reported in Table 5, Table 6 and Table 7, showed that activation control was the strongest predictor of hearing difficulties, whereas attentional focus was the strongest predictor of annoyance and interference from noise. It should be kept in mind that stepwise regression analyses focus on the strongest predictor but do not include the variance shared between all the predictors. Therefore, results from regression analyses should be read in conjunction with results from correlations analyses, which include all temperament variables that are related to each reaction to noise.

### 3.3. Discussion

Study 2 is the first one to study children’s prosocial behaviour and effortful control alongside four reactions to noise: hearing difficulties, attention capture, interference and annoyance from noise. Prosocial behaviour was not related to any of the children’s reactions to noise. Effortful control was related to hearing difficulties, interference and annoyance from noise, but not attention capture. This means that effortful control was related to how children deal with the noise when it is present in the classroom. This is the first study to find differential associations between sub-components of noise sensitivity and effortful control. 

Children who had difficulties in hearing someone talk in the classroom also had lower activation control, attentional focus and inhibitory control. Lower activation control (e.g., the capacity to perform an action when there is a strong tendency to avoid it [57]) was the strongest predictor of hearing difficulties in stepwise regression analyses. In a classroom context, activation control might indicate children’s capacity to orient their attention towards the person who is talking when distractors, such as noise, are present. 

Greater interference from noise was associated with greater impulsivity, lower inhibitory control and lower attentional focus. Lower attentional focus (e.g., the ability to pay attention and stay on task) was the strongest predictor of noise interference in stepwise regressions. Children who are worse at staying on task might be more affected by noise interference. Conversely, interference from noise might be reduced if children have a better ability to stay focused on the task in the first place. 

Greater annoyance from noise was associated with all four effortful control subscales. Attentional focus was the strongest predictor of noise annoyance in stepwise regressions. Children might be particularly annoyed by noise when they have difficulties focusing on their task. 

Effortful control variables predicted between 3.2% and 5.7% of the variance in children’s reactions to noise. This is less than what was reported in a previous study, in which switching skills and mind-wandering explained more than 30% of the variance in noise interference [15]. However, in this previous study, switching skills and mind-wandering were measured via child reports [15], a method that might have brought shared variance between the explanatory variables and the outcomes variables, simply because they were both based on child reports. In contrast, Study 2 used a teacher report of children’s effortful control. Despite the smaller predictive power of effortful control variables, results cross-validate the hypothesis of a link between attentional skills and reactions to noise when using information from different sources. 

Prosocial children were not less noise sensitive to noise, when looking across all four types of reactions to noise. The measure of prosocial behaviour focused on behaviours of helpfulness and kindness towards others, which only partly encompass children’s desire to seek social stimulation. This is a limitation of the current study. Using a global measure of extraversion would have more closely aligned with previous studies [11,32,37]. Another possibility is that it is not extraversion and prosocial behaviour per se which relate to reactions to noise, but the arousal mechanisms underlying them [58]. A more direct measure of arousal is needed to clarify this hypothesis. 

## 4. General Conclusions and Suggestions for Future Research

### 4.1. General Conclusions

Study 1 validated a novel questionnaire assessing children’s reactions to classroom noise, against two measures that are typically used with adults, and have been adapted for children. This validation process showed that there are sub-components of noise sensitivity that are not captured by general (i.e., single-item) measures. Indeed, general noise sensitivity was more related to measures of annoyance than interference from noise. Study 2 offered more insight into the mechanisms underlying children’s reactions to classroom noise by studying inter-individual differences. It showed that those children who are rated as having lower effortful control by their teachers experience more negative attitudes towards noise, and in particular more difficulties hearing someone talk, more interference and more annoyance from noise. Effortful control underlies children’s capacity to adapt flexibly to social demands, in particular to regulate their attention and behaviour [46]. Results from the current study indicate that children with lower effortful control accumulate multiple risks. They are more likely to have difficulties engaging with school work, staying still and focussing, while at the same time being more vulnerable to distractors. This conclusion is consistent with a previous study in which children who were rated by their parents as having more behavioural difficulties were also more sensitive to noise [42]. Study 2 supports the hypothesis of a link between children’s reactions to noise and their effortful control which has only been hinted at in existing theoretical frameworks and child surveys [15]. 

It is interesting to note that studies using subjective reports of children’s reactions to noise [15], and experimental studies assessing children’s academic [59] and creative [60] performance in noisy conditions, come to different conclusions regarding the role of attentional skills in coping with noise. Experimental studies using laboratory tasks of attentional control suggest that its protective role is age and task specific [59,61], and therefore, it is far from being systematic. Studies using subjective measures, such as the current one, suggest that effortful control does predict noise sensitivity in a variety of classroom-based situations. Subjective reports and behavioural measures are therefore complementary and should not be considered interchangeable [62]. 

### 4.2. Limitations and Suggestions for Future Research

One limitation of the current research is that it did not include measures of children’s living environments. Children living in poverty tend to live in more chaotic places (as measured with the Confusion, Hubbub and Order Scale [63,64,65,66]. They experience less familial routine and structure, more crowded houses, a higher exposition to television and to environmental noise [67,68,69]. There is debate as to whether home chaos trains, or on the contrary exhausts, attentional resources [70]. On the one hand, home chaos could lead children to filter out distractions, or shift their attention away from unwanted and unpredictable stimulations. These children might, therefore, be less sensitive to noise in the long term. On the other hand, noise and confusion could exhaust children’s attentional resources. They might have difficulty keeping all the information in mind while focusing on specific inputs or activities [71], and might therefore be particularly sensitive to noise. Current empirical evidence supports the view of greater home chaos being associated with lower attentional resources [63,65,70,72]. However, a systematic investigation of the associations between children’s living environment, attentional resources and noise sensitivity is needed. Indeed, research on adolescents has revealed complex interactions between participants’ age, their living environment and their performance at an attentional inhibitory control task [73].

More work can be conducted to better understand why some children are more sensitive to noise than others. In the current study, the link between extraversion and children’s reactions to noise was not directly tested. Instead, prosocial behaviour was measured as an indicator of children’s engagement in social situations. Using a specific measure of extraversion, instead of prosocial tendencies, would allow for a closer replication of adult findings with child populations. The mechanisms underlying the association between extraversion and more positive attitudes towards noise are still unclear. Two mechanisms in particular deserve further investigation: arousal and participants’ need for privacy. Conducting qualitative interviews with elementary school children would help to better capture the richness of children’s reaction to noise, and of the complex factors influencing them. 

Given the importance of effortful control skills to succeed at school, children at risk of school difficulty might also be the ones who are particularly vulnerable to noise. Interventions aimed at reducing classroom noise [48,74] and/or improving effortful control [55] might be particularly beneficial for the most noise-sensitive children. 

## Figures and Tables

**Table 1:** Descriptive statistics for all the variables used in Study 1.

	*n*	Min	Max	*M*	*SD*	Skewness	Kurtosis
NS (Single item)	103	1	4	1.75	0.87	0.97	0.14
NS (Multiple items)	104	1.29	3.71	2.48	0.60	−0.05	−0.92
Noise levels	104	1	4	2.86	0.65	−0.37	0.12
Hearing Difficulties	104	1	4	1.55	0.74	1.63	2.48
Annoyance	104	1	4	2.23	0.80	0.32	−0.65
Attention Capture	103	1	4	2.26	0.86	0.31	−0.69
Interference	101	1	4	2.11	0.81	0.42	−0.47
Calm	103	1	4	3.18	0.88	−0.90	0.06
Relaxed	102	1	4	2.85	1	−0.33	−1.01
Irritated	102	1	4	1.26	0.63	2.42	5.20

NS: Noise sensitivity.

**Table 2:** Correlations between all the variables used in Study 1.

	1	2	3	4	5	6	7	8	9	10
1. NS (Single item)		.41 ***	.08	.35 ***	.39 ***	.15	.23 *	−.13	.07	−.01
2. NS (Multiple items)	.40 ***		.27 **	.31 **	.61 ***	.40 ***	.43 ***	−.13	−.09	.27 **
3. Noise levels	.08	.27 **		.04	.39 ***	.14	.24 *	.08	.11	.16
4. Hearing Difficulties	.35 ***	.32 **	.04		.31 **	.22 *	.32 **	−.14	−.07	.21 *
5. Annoyance	.39 ***	.61 ***	.39 ***	.31 **		.39 ***	.35 ***	−.02	.12	.25 *
6. Attention Capture	.16	.41 ***	.14	.22 *	.39 ***		.57 ***	−.19	−.08	.26 *
7. Interference	.23 *	.43 ***	.24 *	.33 **	.35 ***	.57 ***		−.13	−.02	.36 ***
8. Calm	−.14	−.16	.08	−.14	−.02	−.18	−.15		.52 ***	−.17
9. Relaxed	.06	−.11	.11	−.07	.12	−.08	−.02	.51 ***		−.13
10. Irritated	−.01	.26 **	.16	.22 *	.26 *	.27 **	.35 ***	−.19	−.15	

Upper triangle: first-order Pearson correlations. Lower triangle: partial correlations controlling for age. NS: Noise Sensitivity; * *p* < .05; ** *p* < .01; *** *p* < .001.

**Table 3:** Descriptive statistics for all the variables used in Study 2.

	*n*	Min	Max	*M*	*SD*	Skewness	Kurtosis
**Reactions to classroom noise**							
Hearing Difficulties	227	1	4	2.06	0.76	0.62	−0.03
Annoyance	223	1	4	2.61	0.90	−0.03	−1.01
Attention Capture	226	1	4	2.57	0.93	−0.05	−1.05
Interference	223	1	4	2.69	0.89	−0.11	−1.06
**Temperament**							
Attentional Focus	230	1	5	3.98	1	−1.03	0.53
Impulsivity	230	1	5	1.96	0.98	0.96	0.15
Inhibitory Control	230	1	5	4.07	0.85	−1.16	1.13
Activation Control	230	1	5	4.06	1	−1.22	0.93
Prosocial Behaviour	230	1.20	3	2.58	0.48	−1.01	−0.07

**Table 4:** Correlations between all the variables used in Study 2.

	1	2	3	4	5	6	7	8	9
1. Hearing Difficulties		.34 ***	.32 ***	.35 ***	−.24 **	.13	−.15 *	−.24 ***	−.13
2. Annoyance	.34 ***		.45 ***	.51 ***	−.21 **	.15 *	−.15 *	−.16 *	−.01
3. Attention Capture	.32 ***	.45 ***		.61 ***	−.09	.12	−.09	−.10	−.01
4. Interference	.35 ***	.51 ***	.61 ***		−.18 *	.15 *	−.15 *	−.13	−.03
5. Attentional Focus	−.24 ***	−.21 **	−.09	−.17 *		−.75 ***	.75 ***	.77 ***	.59 ***
6. Impulsivity	.13	.15 *	.12	.15 *	−.75 ***		−.84 ***	−.64 ***	−.56 ***
7. Inhibitory Control	−.15 *	−.15 *	−.09	−.15 *	.75 ***	−.84 ***		.73 ***	.60 ***
8. Activation Control	−.24 ***	−.16 *	−.10	−.13	.77 ***	−.64 ***	.73 ***		.66 ***
9. Prosocial Behaviour	−.13	−.01	−.01	−.03	.59 ***	−.56 ***	.60 ***	.66 ***	

Upper triangle: first-order Pearson correlations. Lower triangle: partial correlations controlling for age. * *p* < .05; ** *p* < .01; *** *p* < .001.

**Table 5:** Stepwise regression predicting Hearing difficulties from children’s Attentional Focus, Impulsivity, Inhibitory Control and Activation Control, with Age as a control variable (Study 2).

Hearing Difficulties	*β*	*β* (*p*)	R^2^
Age	−.01	.909	
Activation Control	−.24	<.001	
			5.7

*n* = 220.

**Table 6:** Stepwise regression predicting noise Annoyance from children’s Attentional Focus, Impulsivity, Inhibitory Control and Activation Control, with Age as a control variable (Study 2).

Annoyance	*β*	*β* (*p*)	R^2^
Age	−.02	.750	
Attentional Focus	−.21	.002	
			4.5

*n* = 216.

**Table 7:** Stepwise regression predicting noise Interference from children’s Attentional Focus, Impulsivity, Inhibitory Control and Activation Control, with Age as a control variable (Study 2).

Interference	*β*	*β* (*p*)	R^2^
Age	−.04	.587	
Attentional Focus	−.17	.012	
			3.2

*n* = 216.

## Data Availability

The data presented in Study 1 and Study 2 are available on request from the corresponding author. The data are not publicly available due to participants being informed that only averages and group scores would be made public.

## Appendix A

**Table A1:** Full Questionnaire used in Study 1.

**Reactions to Classroom Noise**
*Noise sensitivity (Single item)*
Generally, you are… (1) Not at all noise sensitive, (2) A bit noise sensitive, (3) Rather noise sensitive, (4) Very noise sensitive
*Noise sensitivity (Multiple items, from Weinstein (1978* [11]*)*
At movies, whispering and crinkling candy wrappers disturb me.
At home, I get annoyed when others are noisy.
Sometimes noises get on my nerves and get me irritated.
Even music I normally like will bother me if I’m trying to concentrate.
There are often times when I want complete silence.
I find it hard to relax in a place that’s noisy.
I get mad at people who make noise that keeps me from falling asleep or getting work done.
(1) Not at all true, (2) A bit True, (3) Rather True, (4) Definitely true
*Noise levels in the classroom (from Massonnié et al., 2020* [15]*)*
Do you think your classroom is noisy? (1) Not noisy at all, (2) A bit noisy, (3), Quite noisy, (4) Very noisy
Do you think that the noise level in class is… (1) Very low, (2) Quite low, (3) Quite loud, (4) Very loud
In general, in class, you find your classmates… (1) Not at all noisy, (2) A bit noisy, (3) Quite noisy, (4) Very noisy
*Hearing Difficulties (from Massonnié et al., 2020* [15]*)*
When the teacher, or a classmate talks to the entire classroom… You have difficulties hearing what the person says
When the teacher, or a classmate comes closer to talk to you… You have difficulties hearing what the person tells you
(1) Almost never, (2) Rarely, (3) Quite often, (4) Very often
*Annoyance (from Massonnié et al., 2020* [15]*)*
Are you annoyed by noise in the classroom? (1) Not at all annoyed, (2) A bit annoyed, (3) Quite annoyed, (4) Really annoyed
When the teacher, or a classmate talks to the entire classroom… You are annoyed by noise in the classroom
When the teacher, or a classmate comes closer to talk to you… You are annoyed by noise in the classroom
When you do homework on your own… You are annoyed by noise in the classroom
When you do homework in a group… You are annoyed by noise in the classroom
(1) Almost never, (2) Rarely, (3) Quite often, (4) Very often
*Attention Capture (from Massonnié et al., 2020* [15]*)*
When the teacher, or a classmate talks to the entire classroom… Classroom noise attracts your attention
When the teacher, or a classmate comes closer to talk to you… Classroom noise attracts your attention
When you do homework on your own… Classroom noise attracts your attention
When you do homework in a group… Classroom noise attracts your attention
(1) Almost never, (2) Rarely, (3) Quite often, (4) Very often
*Interference (from Massonnié et al., 2020* [15]*)*
When the teacher, or a classmate talks to the entire classroom… If noise attracts your attention, you lose track of the discussion
When the teacher, or a classmate comes closer to talk to you… If noise attracts your attention, you lose track of the discussion
When you do homework on your own… If noise attracts your attention, you lose track of your thoughts
When you do homework in a group… If noise coming from outside the group attracts your attention, you lose track of the discussion
(1) Almost never, (2) Rarely, (3) Quite often, (4) Very often
**Mood**
Right now, do you feel calm? (1) Not calm at all, (2) A bit calm, (3) Rather calm, (4) Very calm
Right now, do you feel relaxed? (1) Not at all relaxed, (2) A bit relaxed, (3) Rather relaxed, (4) Very relaxed
Right now, do you feel irritated? (1) Not at all irritated, (2) A bit irritated, (3) Rather irritated, (4) Very irritated

## Appendix B

**Table A2:** Reactions to Classroom Noise Questionnaire used in Study 2 (from Massonnié et al., 2020 [15]).

**How Often Does This Happen?**	**Almost** **Never**	**Rarely**	**Quite** **Often**	**Very** **Often**
**When the teacher, or a pupil talks to the entire classroom**				
It’s hard to hear what the person says.	□	□	□	□
You are annoyed by noise in the classroom.	□	□	□	□
Classroom noise catches your attention.	□	□	□	□
If noise catches your attention, you lose track of the discussion.	□	□	□	□
**When the teacher, or a classmate, comes closer to talk to you**				
It’s hard to hear what the person says.	□	□	□	□
You are annoyed by noise in the classroom.	□	□	□	□
Classroom noise catches your attention.	□	□	□	□
If noise catches your attention, you lose track of the discussion.	□	□	□	□
**When you do an activity alone, in the classroom**				
You are annoyed by noise in the classroom.	□	□	□	□
Classroom noise catches your attention.	□	□	□	□
If noise catches your attention, you lose track of your thoughts.	□	□	□	□
**When you are doing a group activity**				
You are annoyed by noise in the classroom.	□	□	□	□
Noise from outside the group catches your attention.	□	□	□	□
If noise coming from outside the group catches your attention, you lose track of the discussion.	□	□	□	□
